# Water Regime and Nitrogen Management to Cope with Wheat Yield Variability under the Mediterranean Conditions of Southern Portugal

**DOI:** 10.3390/plants8100429

**Published:** 2019-10-19

**Authors:** Manuel Patanita, Alexandra Tomaz, Tiago Ramos, Patrícia Oliveira, Luís Boteta, José Dôres

**Affiliations:** 1 Escola Superior Agrária - Instituto Politécnico de Beja, R. Pedro Soares S/N, 7800-295 Beja, Portugal; mpatanita@ipbeja.pt (M.P.);; 2 GeoBioTec, Universidade Nova de Lisboa, Campus da Caparica, 2829-516 Caparica, Portugal; 3 Centro Operativo e de Tecnologia de Regadio, Quinta da Saúde, 7801-904 Apartado Beja 354, Portugal

**Keywords:** wheat, climate change, climate uncertainty, grain yield, irrigation, nitrogen fertilization, enhanced efficiency fertilizers

## Abstract

Global climate change accentuates the seasonal and interannual irregularity of temperature and precipitation of the Mediterranean climate. The consequences of this variability on wheat production are felt on its development cycle and productivity, making the production chain of this crop vulnerable to the occurrence of years with abnormal distributions of precipitation and with extreme temperatures. Adaptation strategies like irrigation or fertilization can help to cope with the negative impacts of climate uncertainty. This study evaluated the effects of water regime and nitrogen (N) fertilization techniques on wheat production in southern Portugal based on the results of three trials conducted in two agricultural years (2016/2017 and 2017/2018) with contrasting climate conditions. Phenology and yield were evaluated by comparing water regimes (R1, full irrigation; R2, supplemental irrigation at four stages: start of stem extension, booting, anthesis, grain filling; R0, rainfed (in 2017/2018)) and N fertilization splitting/timing and type (conventional and enhanced efficiency fertilizers (EEFs): controlled-release N, stabilized with nitrification inhibitor, and stabilized with urease inhibitor). Significant effects of water regime on grain yield were obtained in 2016/2017, a year with extreme aridity and high water requirements felt from the tillering stage, in the trial with conventional fertilizers. In 2017/2018, when a beneficial seasonal rainfall distribution occurred, water regime did not influence grain yield, pointing to the feasibility of supplementary irrigation to maximize water productivity. Nitrogen fertilization influenced yield and its components, with the highest values of grain yield being obtained with conventional fertilizer. Regardless of the possible effects on grain quality, the use of EEF did not prove to have an indisputable effect on wheat yield in the conditions under which the trials were conducted. Comparison of the results in the two years accentuates the need to continue the evaluation of the influence of agronomic management in wheat production in the context of adaptation to the climatic uncertainty in Mediterranean regions.

## 1. Introduction

According to [1], the contribution of Portugal to common wheat (*Triticum aestivum* L.) production in the EU-28 represented in 2017 only 0.04% in a production area of 0.11%. Therefore, Portugal is an importer of common wheat, and this situation is difficult to overcome given the market fluctuations and the less than optimal Mediterranean climate conditions for wheat production, particularly high rainfall irregularity and growing drought trends caused by climate change [2,3,4].

The Mediterranean and other South European regions are especially vulnerable to climate change, facing increased competition for water resources between different sectors (agriculture, industry, or domestic uses) [4]. Climate change accentuates the seasonal and interannual irregularity of temperature and precipitation, traits of the Mediterranean climate, making periods with high temperatures and water limitations more pronounced [2,3,4]. The projections for the Mediterranean area are a gradual increase of temperature and a decrease in rainfall. Moreover, an increase in the frequency and magnitude of extreme events of heat waves is also predicted [2,3,4,5,6]. These trends will promote sharp declines in the production of rainfed crops, leading to the escalation in irrigation needs [4,7]. Under these conditions, wheat (*Triticum aestivum* L.) is one of the crops that will suffer the greatest reduction in productivity as a result of the expected extreme environments [8,9]. The consequences of this climatic uncertainty in Mediterranean environments on wheat production will potentially lead to yield losses that may reach one-third of the current value [8].

In Mediterranean regions, the interannual yields of wheat are irregular and influenced by water availability and heat stress, and their occurrence in certain periods of the development cycle [5,6]. Sensitivity to high temperatures is higher during reproductive stages than in vegetative stages [10], the most sensitive periods being anthesis and grain filling [11,12,13]. According to [14], heat stress, as well as limited water availability, can significantly reduce the rate of photosynthesis, reducing the amount of assimilates available to the grain, therefore affecting mean grain weight and water use efficiency (WUE) [15,16]. Other stages, like stem elongation and booting, are reported as being susceptible to water stress due to reductions in potential grain number per unit area [16,17,18,19]. Furthermore, water shortages combined with nitrogen (N) deficiency can also concur with the reduction in grain number [16,20]. In fact, crop responses to N depend on available water in soil, rainfall amount, and distribution during the growth cycle [21,22]. Grain yield and N use efficiency (NUE) decrease under water deficit conditions and elevated temperatures, particularly if they occur around anthesis [16,23,24]. Nitrogen content is widely considered as the main factor that affects storage proteins and grain quality in wheat [25]. Therefore, the productivity and grain quality of wheat in response to N availability is also dependent on growth stage. Authors in [26] state that approximately 40% to 90% of grain nitrogen in wheat originates from the remobilization of N stored in vegetative tissues before anthesis, so that N remobilization depends on these nutrient sources.

Strategies to increase NUE include management practices, like rate, time, or method of application, and the development of new technologies, like the use of alternative fertilization techniques with the so-called enhanced efficiency fertilizers (EEFs) [27,28]. These kinds of fertilizers that delay the bioavailability of nitrogen in the soil, matching its release with the crops’ higher needs periods, are classified as [29]: (i) slow-release fertilizers (obtained as condensation products of urea and urea aldehydes); (ii) controlled-release fertilizers (products containing a conventional fertilizer whose nutrient release in the soil is regulated by sulfur or/and polymer coatings); (iii) stabilized fertilizers (which are modified during the production process with a nitrification inhibitor or an urease inhibitor). Several studies have shown that the use of such fertilizers has been successful in nurseries [30] or in conditions of high rainfall and in sandy soils [28]. In irrigated crops, where N fertilizers are partially applied through the irrigation water, EFFs have the potential to contribute to the increase of resource-use efficiency by promoting higher yields, higher grain quality, and reducing leaching risks [28,31,32]. 

The overlapping of key climate variables and the critical stages of the wheat development cycle, along with the climate uncertainty associated with Mediterranean conditions, implies that the success of the crop depends, to a large extent, on the combination of appropriate management strategies. Adaptation to climate change and climatic variability must take place through introduction of short-run field adjustments and/or long-term adaptations [5,8,15,33,34,35]. While the latter refer to major structural transitions involving changes in land allocation, substitution of crops, or breeding of crop cultivars [8,15], the former include efforts to optimize production without major system changes, such as use of species and/or cultivars resistant to heat and drought, alteration of sowing dates and planting densities, improved irrigation techniques, improved fertilizers use, and other different soil or crop management practices (e.g., mulching, crop rotation, intercropping) [36,37,38,39,40,41]. In wheat production, providing water through supplemental irrigation and appropriate rate and time of N fertilizer application can be integral to stabilize yields, increase productivity, and to enhance the industrial quality of the grain [19,25,26,42,43]. 

Many crop simulation studies have been conducted recently using climate change scenarios and cropping systems models to estimate crop productivity under different agronomic practices [9,34,44,45,46]. However, field studies designed to evaluate wheat adaptation to optimized irrigation or fertilization are also very important. These studies should aim to contribute to the increase of WUE and NUE and, thus, to the strengthening and adaptation of the wheat production chain under the typical variability of Mediterranean climate.

Taking the above into consideration, this study aimed to evaluate the effects of water regime and nitrogen fertilization type/splitting/timing under the Mediterranean environment of southern Portugal on common wheat phenology, yield, and yield components. Understanding whether the use of supplemental irrigation and “special N fertilizers”, as opposed to water comfort irrigation and conventional fertilizers as technical options that will maximize productivity, may contribute to the selection of more suitable agronomic practices. These options could allow the stabilization and maximization of wheat production, adjusting to the typical constraints and risks of the climatic uncertain conditions of the Mediterranean regions. 

## 2. Results and Discussion

### 2.1. Climate, Irrigation, and Phenology

**Figure 1:**
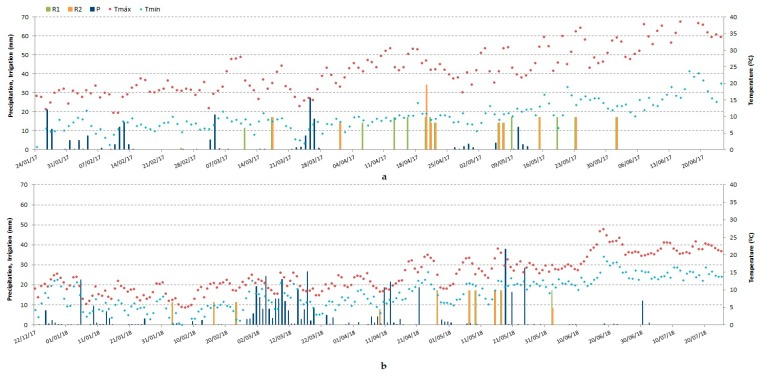
Irrigation depths (R1 and R2), precipitation (P), maximum daily temperature (Tmax), and minimum daily temperature (Tmin) in Beja. **a**-2016/2017 crop cycle; **b**-2017/2018 crop cycle. R1, 100% of crop evapotranspiration (ETc); R2, 100% of ETc at the critical stages of beginning of stem extension, booting, heading, and grain filling.

2016/2017 and 2017/2018 were paradigmatic examples of the climatic uncertainty characteristic of southern Portugal. The average of the maximum temperatures recorded differed by 10 °C in the two wheat growth cycles (Figure 1). The spring of 2017 registered several daily temperature peaks above 35 °C since May. In fact, the year 2017 in Portugal was classified as extremely hot and dry, being the third driest and the second warmest year since 1931 [47]. Through the year, there were long periods of high temperatures and low precipitation so that by the end of October, mainland Portugal was under extreme drought, the severest class according to the Palmer drought severity index (PDSI) [48]. These conditions gave rise to extreme aridity, and high water requirements were felt from the beginning of March, when crops were entering the tillering stage, until the end of the crop cycle (Figure 1). In the R1 treatment (full irrigation with 100% of crop evapotranspiration (ETc) throughout the cycle), the first irrigation took place on 11 March 2017, while in R2 (supplemental irrigation with 100% of ETc at four stages: beginning of stem extension, booting, heading, and grain filling), irrigation began on 17 March 2017. Irrigation became more frequent after April, as temperature and evapotranspiration increased. As defined in the schedule criteria of the treatments, R1 irrigation aimed at replenishing the total soil water storage capacity every time the soil water balance showed an oncoming water deficit, with intervals of 2 to 15 d; in R2, irrigation took place every 15–20 d until May. At flowering and grain filling, given the increased water requirements of the crops in these stages, irrigation was applied weekly.

In 2017/2018, the cumulative rainfall during the wheat development cycle was 291 mm higher than in the previous year, mostly concentrated during the spring. Therefore, there were considerable differences in irrigation needs in the two years: in the full irrigation water regime, R1 (100% of crop evapotranspiration (ETc) throughout the growing cycle), the total irrigation volumes in 2016/2017 and 2017/2018 were 2527 and 1440 m^3^ ha^−1^, respectively. In other words, there was a difference of 1087 m^3^ ha^−1^ between the irrigation requirements in the two crop cycles. 

In 2016/2017, the total irrigation volume in the full irrigation treatment (R1) was 804 m^3^ ha^−1^ higher than the volume applied in supplemental irrigation treatment (R2). In contrast, in 2017/2018, as a result of the abundant spring rains coincident with some of the phases where wheat had greater water sensitivity (such as booting or grain filling), the difference between R1 and R2 was only of 80 m^3^ ha^−1^, about 90% less than the preceding year. Therefore, in the 2017/2018 growth cycle, the availability of soil water provided by the spring precipitation distribution meant that a true differentiation between the volumes and irrigation dates in the R1 and R2 water regimes was not possible. The total number of irrigation events was 10, and the first and last irrigations took place, respectively, on 03/02/2018 and on 02/06/2018, with an average interval of 13 d. This implied that the phenological phases in different irrigation treatments occurred generally on very similar dates (Table 1). Although there were different sowing dates in the trials conducted in the two years, it is possible that the high temperatures felt in the spring of 2016/2017 were an additional cause for the difference in the phenology progress, with the end of the crop cycle in 2016/2017 being anticipated 1 month as a result of a shorter grain filling and ripening period (from heading to harvest). 

**Figure 2:**
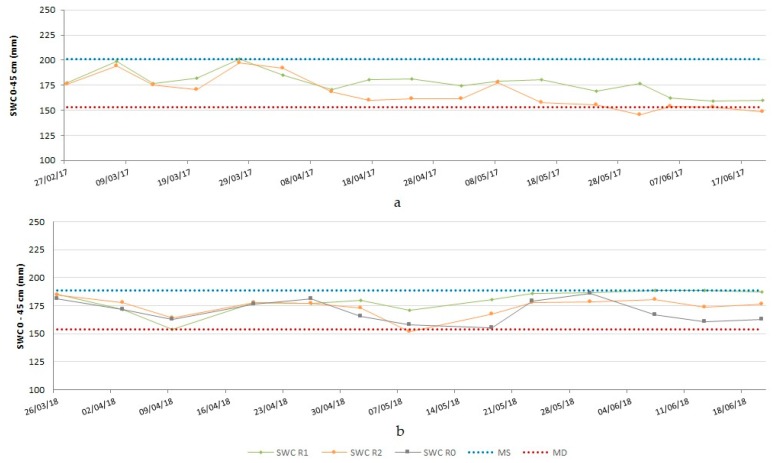
Soil water content (SWC; mm of water in the 0–45 cm soil profile) in the different water regime treatments. **a**-2016/2017 crop cycle; **b**-2017/2018 crop cycle. R1, irrigation with 100% of ETc; R2, irrigation with 100% of ETc at critical stages of beginning of stem extension, booting, heading, and grain filling. MS, maximum storage during the growth cycle (mm of water in the 0–45 cm soil profile); MD, maximum allowed depletion during the growth cycle (mm of water in the 0–45 cm soil profile).

The temporal evolution of soil water content in the different irrigation regimes tested in both years can be observed in Figure 2. In 2016/2017, despite the physical properties of the soils in the study area, like high water-holding capacity, a trait of vertisols, as the season advanced, soil water content (SWC) in the supplemental irrigation water regime (R2) only approached SWC in R1 whenever irrigation was applied; furthermore, SWC in R2 decreased with time, at times reaching values below the offset of crop stress (beneath maximum depletion (MD)) in the final stages of the growth cycle. 

In 2017/2018, SWC was influenced not only by the irrigation applied but also by the occurrence of late spring precipitation and by the high water-holding capacity of the soil, leading to periods with a growing trend in available soil water, even in the rainfed treatment, in the final stages of the crop growth cycle. The offset of crop water stress was momentarily reached in early May in the supplemental irrigation plots.

### 2.2. Grain Yield and Yield Components

Interannual and seasonal climatic variability, well evidenced in these two years, can have implications either at the phenology level—with cycles of different duration, fruit of the anticipation, or delay in the final phenological states—or at yield [6,9] and even at the level of grain [18,49]. For example, looking at the average yields in each of the trials (Table 2 and Table 3), in 2016/2017, average values were 4268 and 4551 kg ha^−1^, respectively, in Trial 1 (to evaluate the effect of two irrigation regimes, R1 and R2, and six nitrogen fertilization treatments of splitting/timing with enhanced efficiency N fertilizers, E1 to E6, on grain yield and grain yield components) and Trial 2 (to evaluate the effect of two irrigation regimes, R1 and R2, and five nitrogen fertilization treatments of splitting/timing with conventional N fertilizers, C1 to C5, on grain yield and grain yield components), while in the 2017/2018 experiment (to evaluate the effect of three irrigation regimes—R1, R2, and rainfed (R0)—and eight nitrogen fertilization treatments of type/splitting/timing with enhanced efficiency N fertilizers and conventional fertilizers, N1 to N8, on grain yield and grain yield components), the average yield reached 7100 kg ha^−1^, which was, respectively, 1.7 and 1.6 times higher than the previous years.

In Trial 1, only the number of spikes per m^−2^ showed significant influence of the irrigation regime, the highest value being registered in the R1 irrigation regime (396 spikes per m^−2^) (Table 2a). These results, with no significant differences in wheat yield between water regimes, may point to a greater efficiency in irrigation water use in the irrigation strategy, R2, suggesting that supplemental water applied at the defined critical periods is used more efficiently by the crop. These results are in agreement with [38] where, when studying different supplemental irrigation strategies at different growth stages and application of different rates of nitrogen fertilizer on yield and water productivity (WP) of wheat cultivars, they reported improved yield and WP when using supplemental irrigation at the beginning of stem elongation. There was a significant effect of N fertilization in yield, the highest value being obtained in the 75% sowing +25% stem extension splitting/timing (E5) at 4564 kg ha^−1^. This result indicates that early N applications with this type of fertilizer does not compromise N availability throughout the wheat growth cycle and, therefore, the grain production. Nevertheless, it is important to ascertain whether the N availability throughout the wheat cycle that these ‘special’ fertilizers seemingly provide is also suitable for a fair quality of grain and flour.

In Trial 2, with conventional N fertilizer, significantly higher 1000-grain weights (41.56 g) and yield (5614 kg ha^−1^) were obtained in the R1 treatment (Table 2b), a result in accordance with [23] that observed a significant, positive effect of irrigation in comparison with no irrigation, in wheat grain yield and root weight density. Similar results were found by [39] when comparing two irrigation strategies with rainfed condition in winter wheat produced in the North China Plain. No significant effect of N fertilization was determined in Trial 2. No interaction between factors (water regime × N fertilizer splitting/timing) was felt in yield and its components.

The year 2017/2018 was characterized by a beneficial distribution of precipitation for wheat development (Figure 1). There was a statistically significant effect of water regime both in the number of grains per m^−2^ and 1000-grain weight (g), showing a compensation effect in these yield components that lead to no statistical differences in grain yield (Table 3). Authors in [16] in a study on the interactive effects of water and nitrogen on durum wheat (*Triticum durum* Desf.) grown in a Mediterranean environment found similar results, with the crop response being mostly influenced by nitrogen fertilization as a consequence of the occurrence of abundant rainfall during the experiment period.

The effect of the water regime on the main yield components seemed to be stronger on the 1000-grain weight, since the values obtained were statistically different in the three treatments, with R1 having the advantage (47.57 g) followed by R2 (46.14 g). The occurrence of precipitation throughout the crop cycle, favorably distributed and concentrated during heading and initial grain filling, attenuated and/or eliminated the differences between the different water regimes and highlights the influence of water supply during the grain filling stage. This attenuating effect is even more pronounced when soils present a high water storage capacity, as is the case of vertisols (Figure 2).

Nitrogen fertilizer splitting/timing had no significant effect on the number of spikes (m^−2^). Regarding the effect of nitrogen fertilization on yield components, the results were as follows: (i) there was an effect on the number of grains, with the highest values occurring in the treatments with conventional fertilizer, N1 (16,158 grains per m^−2^) and N2 (16,145 grains per m^−2^), and the lowest value registered in the N5 treatment (15,091 grains per m^−2^) with controlled-release fertilizer; (ii) the N5 treatment also presented the lowest grain weight value (45.13 g); (iii) N1 and N2 were the nitrogen fertilizer treatments with the highest yields (7378 and 7337 Kg ha^−1^, respectively). This set of results in 2017/2018 indicates that the use of ”special” fertilizers, as opposed to using conventional fertilizers, had no distinguishing effect on wheat productivity, and it is in accordance to [25] and [31], where the application of slow-release and controlled-release polymer-coated nitrogen fertilizers, respectively, in wheat and maize (*Zea mays L*.) resulted in no observed differences in grain yield. Additionally, when comparing treatments with the same type of fertilizer, results indicate a positive effect on wheat yield resulting from fertilizer splitting in the case of controlled-release (N5 and N6) and urease inhibitor (N7 and N8) fertilizer. In the case of the nitrification inhibitor fertilizer, a higher yield was obtained in the treatment where total N was applied at sowing (N3, when comparing with N4). No interaction water regime × N splitting/timing was felt in yield and its components.

## 3. Materials and Methods 

### 3.1. Site Description

The study took place during the agricultural years 2016/2017 and 2017/2018 in Beja (Baixo Alentejo, Southern Portugal) with the cultivar of common wheat Antequera, classified as “improver” by the milling industry and included in the list of recommended varieties of common wheat in both agricultural years [51].

The climate in the study area is Mediterranean (Csa, in Köppen classification) with climate normals (1981–2010) for annual precipitation and average mean daily temperature of, respectively, 558 mm and 16.9 °C [52]. Soils are predominantly pellic vertisols associated with calcic cambisols [53,54,55]; that is, they are heavy-textured soils with high moisture-holding capacity and possible accumulation of secondary carbonates.

Meteorological data were recorded in an automatic weather station belonging to the Agrometeorological System for Irrigation Management in Alentejo region (SAGRA-Sistema Agrometeorológico para a Gestão da Rega no Alentejo, [56]). 

Irrigation was performed by a center-pivot system. The irrigation amounts and schedules were evaluated using the Irrigation Management Model for the Alentejo region (MOGRA—Modelo de Gestão da Rega para o Alentejo, [57]) This model performs daily soil water balancing, based on the FAO methodology for computing crop water requirements [58], using meteorological data, the crop’s specific information, and soil water content (SWC) data registered in the main plots with capacitance probes (PR1 Profile Probe, Delta-T Devices, Ltd.) with 45 cm depth and four sensors with a 10 cm step. In order to evaluate the soil’s available water dynamics, continuous monitoring capacitance probes (Enviroscan, Sentek Technologies, Ltd.) were also installed each year in the main plots.

### 3.2. Study Design

#### 3.2.1. Trials in 2016/2017

Two trials (Trial 1 and Trial 2) were carried out during the 2016/2017 agricultural year. In both trials, wheat was sown on 24 January 2017 and harvested on 24 June 2017. The experimental design was split-plot with two irrigation treatments as main plots: R1, full irrigation with 100% of crop evapotranspiration (ETc) throughout the cycle; R2, supplemental irrigation with 100% of ETc only at four critical stages (according to the decimal code of the Zadoks scale [59]: 30, beginning of stem extension; 40 to 49, booting; 50 to 59, heading or inflorescence emergence; 70 to 89, grain filling). The total irrigation volumes during the 2017 crop cycle were 2527 and 1723 m^3^ ha^−1^, respectively, in R1 and R2 treatments. 

The subplots (9.6 m^2^) were N fertilizer splitting and timing of application treatments. More specifically, plots included six treatments in Trial 1, with enhanced efficiency fertilizers applied at sowing-stabilized (with the nitrification inhibitor DMPP (3,4-phosphate dimetilpyrazol)) (E1 to E5) and controlled-release (i.e., with a polymer that coats the fertilizer granules, protecting nutrients from leaching losses, and ensuring their availability for plant uptake throughout the cycle) (E6), with three replications (Table 4); and five treatments in Trial 2 (C1 to C5), with conventional N fertilizer with three replications (Table 5). The applied N fertilizer dose was 165 kg N ha^−1^, following the recommendations of the Ordinance 259/2012 [60] that establishes the Portuguese action program for vulnerable areas to nitrates pollution caused by agricultural practices (implemented after the Decree-Law 235/97 [61] that transposes into national law the EEC Council Directive 91/676 [62] concerning the protection of water against pollution caused by nitrates from agricultural sources). According to [60], for a potential wheat yield of 5 ton ha^−1^, an application rate of 165 N ha^−1^ is recommended. To ensure equal phosphorus (P) and potassium (K) rates in all treatments, a binary P-K fertilizer (Amicote CV 44 0-27–17) was applied at sowing. Topdressing N fertilization was applied at tillering, with urea (Ureia 46%), and with ammonium nitrate (Nitrolusal 27%) at the remaining stages.

#### 3.2.2. Trial in 2017/2018

The experimental design was split-plot with three water regime treatments as main plots: R0, rainfed; R1 and R2, as described in Section 3.1. The subplots, with four replications, were the nitrogen (N) fertilizer type used at sowing, splitting (% of N total), and timing (phenological stage) treatments (Table 6). The total dose applied was 180 kg N ha^−1^, following the recommendations of [60] for a wheat expected yield of 5.5 ton ha^−1^ in the study area (due to the earliest sowing date, an expected yield increase of 0.5 ton ha^−1^ was considered, and the fertilization rate was adjusted). Fertilization treatments were N1 and N2, conventional fertilizer; N3 and N4, stabilized fertilizer (with nitrification inhibitor); N5 and N6, controlled-release fertilizer (polymer coating); N7 and N8, stabilized fertilizer (with urease inhibitor MCDHS (mono carbamide dihydrogen sulphate), a chemical additive that acts on urease, inhibiting the transformation of urea nitrogen into ammonia nitrogen). Each pair is distinguished by N splitting over the crop cycle. In N treatments numbered with even numbers, topdressing N fertilization was applied with urea (Ureia 46%) and ammonium nitrate (Nitrolusal 27%), respectively, at tillering and/or at stem extension and booting stages to ensure the 180 Kg ha^−1^ rate of N fertilization. As described in Section 3.2.1., a binary P–K fertilizer (Amicote CV 44 0–27–17) was applied at sowing to warrant a fertilization rate equivalence between treatments.

Wheat was sown on 22 December 2017, and the harvest took place between 18–25 July 2018. The total irrigation volumes applied during the growth cycle were 1440 and 1350 m^3^ ha^−1^, in treatments R1 and R2, respectively, distributed thought 10 irrigations beginning on 03 February 2018 and ending on 02 June 2018.

### 3.3. Soil Water Content 

Based on the soil water volumetric content (θ_V_) registered by the capacitance probes, the soil water content (SWC; mm) in each day of monitoring was computed using:

(1) $$SWC_{z,i}=\theta v_{z,i}\cdot z,$$

where *SWC_z,i_* is the available soil water in each soil layer on day *i* (mm); *θv_z,,i_* is the soil water volumetric content in each soil layer on day *i*; and *z* is the depth (in mm) of the soil layer covered by the sensor (15 cm in the first layer, in order to take into account the superficial layer from 0 to 5 cm plus the 10 cm diameter of the sensor range, and 10 cm in the remaining three sensors).

The total SWC in the 0–45 cm profile on each day of monitoring, *SWC_i_*, is the sum of the *SWC_z,i_* of the *n* layers covered by the probe (in this case, *n* = 4):

(2) $$SWC_{i}=\overset{n}{\underset{z=1}{\sum }}SWC_{z,i}$$

Each year, the maximum storage (MS) and maximum depletion (MD) were evaluated from the values of *SWC_i_* throughout the cycle to evaluate readily available water (RAW) [58]. In this case:

(3) RAW=MS−MD,

where MS corresponds to field capacity (mm), and MD is the management-allowed depletion or lower threshold of soil water content below which water stress develops (mm). MS and MD were obtained based on the methodology described in [63,64] through observation of the soil water dynamics during each year in the full irrigation treatment (R1) plots. Field capacity is the steady SWC value recorded 24 (light textured soils) to 48 h (heavy textured soils) after probe installation and all gravitational water is drained from the soil, while the offset of crop water stress occurs when there is a marked reduction in the slope of the soil water content curve, denoting the difficulties experienced by plants in extracting water from the soil. Values found for the 0–45 cm soil profile were: MS = 201 mm and MD = 153 mm, in 2016/2017; and MS = 189 mm and MD = 154 mm, in 2017/2018.

### 3.4. Phenology and Yield Evaluation

The main phenological stages in each water regime treatment were registered throughout the crop cycle. Yield and yield components evaluated were grain yield (kg ha^−1^), obtained in each sublot, corrected to 12% moisture, and extrapolated to the hectare; number of spikes per m^−2^, obtained by counting in two areas of 0.2 m^2^ in each subplot and extrapolating to the square meter; 1000-grain weight (g), obtained by electronic counting on a seed counter (Pfeuffer GmbH) of 100 grains, according to ISO 520:1977, followed by weighing and multiplication by 10; and number of grains per m^−2^, determined from dividing grain yield (kg ha^−1^) by 1000-grain weight (g), multiplied by 100.

For the statistical analysis of the data (Analytical Software Statistix 8.0.), a two-way ANOVA was performed (water regime and nitrogen fertilization). Differences between means were compared using Tukey’s test (*p* < 0.05).

## 4. Conclusions

The results highlight the determining influence of the climatic variability typical of the Mediterranean climate on the agronomic yield of common wheat. The extreme aridity and high water requirements felt in 2016/2017 resulted in lower yields and in a differentiation between irrigation treatments in the trial with conventional fertilizer. More precisely, significantly higher values were observed in the full irrigation regime (R1), both in grain weight and in grain yield. However, in the EEF trial, no significant differences between water regimes were observed in grain yield.

The availability of soil water provided by the spring precipitation distribution in 2017/2018, coupled with the large capacity of soil water storage, meant that a true differentiation between the volumes and dates of irrigation in the irrigated treatments was not possible. In this year, the water regime did not influence grain yield, with statistically similar values for the rainfed and the two irrigation regimes. Also, the highest values of grain yield were obtained in treatments with conventional fertilizers indicating that, not considering the possible effects on grain quality, the use of ‘special’ fertilizers had no positive effect on wheat productivity. 

## Figures and Tables

**Table 1:** Phenological states and their dates in the different water regimes in the 2016/2017 and 2017/2018 trials.

Wheat Phenological Stages	2016/2017	2017/2018		
	R1 and R2	R0	R1	R2
Sowing	24/01	22/12	22/12	22/12
Emergence	05/02	03/01	03/01	03/01
Tillering	01/03	15/02	15/02	15/02
Stem elongation	25/03	07/03	10/03	07/03
Booting	14/04	09/04	09/04	09/04
Heading	24/04	24/04	22/04	24/04
*Harvest*	*23/06*	*25/07*	*25/07*	*18/07*
Crop cycle (d)	153	216	214	209

R0, rainfed; R1, irrigation with 100% of ETc throughout the cycle; R2, irrigation with 100% of ETc at beginning of stem extension, booting, heading, and grain filling.

**Table 2:** Effect of the water regime and nitrogen fertilizer splitting/timing on number of spikes per m^−2^, 1000-grains weight (g) and yield (kg ha^−1^; corrected to 12% moisture) with Enhanced Efficiency N fertilizers (Trial 1) and with Conventional N fertilizers (Trial 2) in 2016/2017 (adapted from [50]).

a-Trial 1 (2016/2017)				b-Trial 2 (2016/2017)			
Source of Variation	Number of Spikes per m^−2^	1000-Grains Weight (g)	Yield (kg ha^−1^)	Source of Variation	Number of Spikes per m^−2^	1000-Grains Weight (g)	Yield (kg ha^−1^)
**Water regime**	*	N.s.	N.s.	**Water regime**	N.s.	*	*
**R1**	396 a	42.52	4594	**R1**	393	41.56 a	5614 a
**R2**	354 b	40.03	3942	**R2**	371	39.00 b	3488 b
**N splitting/timing**	N.s.	N.s.	*	**N splitting/timing**	N.s.	N.s.	N.s.
**E1**	335	41.04	4170 ab	**C1**	400	40.05	4694
**E2**	397	42.44	3929 b	**C2**	390	40.79	4688
**E3**	373	40.67	4126 ab	**C3**	386	40.99	4686
**E4**	373	42.14	4458 ab	**C4**	381	38.71	4535
**E5**	400	41.66	4564 a	**C5**	354	40.86	4154
**E6**	371	39.71	4361 ab	**-**	_	_	_
**Interaction**	N.s.	N.s.	N.s.	**Interaction**	N.s.	N.s.	N.s.
**General average**	375	41.28	4268	**General average**	382	40.28	4551

Different letters indicate statistically significant differences (*p* < 0.05) by the Tukey test; *, significance for *p* < 0.05; N.s., no significance for *p* < 0.05. R1, 100% of ETc throughout the cycle; R2, 100% of ETc at stages beginning of stem extension, booting, heading, and grain filling. E1 to E5, stabilized (with nitrification inhibitor) fertilizer splitting/timing treatments; E6, controlled-release (polymer coating) fertilizer splitting/timing treatment (Table 2a). C1 to C5, conventional N fertilizers splitting/timing treatment.

**Table 3:** Effect of the water regime and nitrogen fertilizer type/splitting/timing on number of spikes per m^−2^, number of grains per m^−2^, 1000-grain weight (g), and yield (kg ha^−1^; corrected to 12% moisture) in 2017/2018.

Source of Variation	Number of Spikes per m^−2^	Number of Grains per m^−2^	1000-grain Weight (g)	Yield (kg ha^−1^)
**Water regime** **R0** **R1** **R2**	N.s. 511 467 463	* 16182 a 15814 ab 14583 b	* 43.82 c 47.57 a 46.14 b	N.s. 7083 7286 6932
**N splitting/timing** **N1** **N2** **N3** **N4** **N5** **N6** **N7** **N8**	N.s. 460 490 510 476 461 477 496 475	* 16158 a 16145 a 15991 ab 15236 ab 15091 b 15138 ab 15219 ab 15242 ab	* 45,72 ab 45,51 ab 45,42 ab 46,69 a 45,13 b 46,33 ab 45,27 ab 46,66 a	* 7378 a 7337 a 7244 ab 7091 abc 6793 c 6999 abc 6873 bc 7089 abc
**Interaction**	N.s.	N.s.	N.s.	N.s.
**General average**	310	15527	45.84	7100

Different letters indicate statistically significant differences (*p* < 0.05) by the Tukey test; *, significance for *p* < 0.05; N.s., no significance for *p* < 0.05. R0, rainfed; R1, 100% of ETc throughout the cycle; R2, 100% of ETc at the stages of beginning of stem extension, booting, heading, and grain filling. N1 and N2, conventional fertilizer; N3 and N4, stabilized (with nitrification inhibitor) fertilizer; N5 and N6, controlled-release fertilizer (polymer coating); N7 and N8, stabilized (with urease inhibitor) fertilizer. Each pair is distinguished by N splitting over the crop cycle.

**Table 4:** Nitrogen fertilizer type, splitting (% of N total) and timing (phenological stage) treatments through the wheat cycle in Trial 1 (2016/2017), with Enhanced Efficiency N fertilizers. Crop stages dates between brackets. N – Nitrogen; P – Phosphorus; K – Potassium.

Treatment (N Type/Splitting/Timing)	Type of Fertilizer at Sowing (Name and NPK rating)	% N total				
		Sowing	Tillering	Stem Extension	Booting	Heading
**E1**	Stabilized, with nitrification inhibitor (*Entec 20-10-10*)	100				
**E2**		50			50	
**E3**		50		25		25
**E4**		75			25	
**E5**		75		25		
**E6**	Controlled release, with polymer coating (*Nergetic 20-8-6*)	100				
Top dressing N fertilizer	-	-	-	Ammonium nitrate (*Nitrolusal 27%*)		

**Table 5:** Nitrogen fertilizer splitting (% of N total) and timing (phenological stage) treatments through the wheat cycle in Trial 2 (2016/2017), with Conventional N fertilizers. Crop stages dates between brackets.

Treatment (N Splitting/Timing)	Type of Fertilizer at Sowing (Name and NPK rating)	% N total				
		Sowing	Tillering	Stem Extension	Booting	Heading
**C1**	Conventional (*Foskamonio 12–24–12*)	33	33	33		
**C2**		25	25	25		25
**C3**		25	25	25	25	
**C4**			50		25	25
**C5**		50		25	25	
Top dressing N fertilizer	-	-	Urea (*Ureia 46%*)	Ammonium nitrate (*Nitrolusal 27%*)		

**Table 6:** Nitrogen fertilizer type and name, splitting (% of N total), and timing (phenological stage) treatments through the wheat cycle.

Treatment (N Type/Splitting/Timing)	Type of Fertilizer at Sowing (Name and NPK rating)	% of N Total Applied at Phenological Stages			
		Sowing	Tillering	Stem Extension	Booting
**N1**	Conventional (Foskamonio 12–24–12)	25	50		25
**N2**		25	25	25	25
**N3**	Stabilized, with nitrification inhibitor (Entec 20–10–10)	100			
**N4**		50			50
**N5**	Controlled release, with polymer coating (Nergetic 20–8–6)	100			
**N6**		50			50
**N7**	Stabilized, with urease inhibitor (Renovation Fuerza Plus 20–5–5)	100			
**N8**		50			50
**Top dressing N fertilizer**	-	-	Urea (Ureia 46%)	Ammonium nitrate (Nitrolusal 27%)	

## References

[1] EUROSTAT (2019). Wheat and Spelt by Area, Production and Humidity.

[2] Páscoa P., Gouveia C.M., Russo A., Trigo R.M. (2017). Drought Trends in the Iberian Peninsula over the Last 112 Years. Adv. Meteorol..

[3] Vicente-Serrano S.M., Lopez-Moreno J.-I., Beguería S., Lorenzo-Lacruz J., Sanchez-Lorenzo A., García-Ruiz J.M., Azorin-Molina C., Morán-Tejeda E., Revuelto J., Trigo R. (2014). Evidence of increasing drought severity caused by temperature rise in southern Europe. Env. Res. Lett..

[4] Kovats R.S., Valentini R., Bouwer L.M., Georgopoulou E., Jacob D., Martin E., Rounsevell M., Soussana J.F., Barros V.R., Field C.B., Dokken D.J., Mastrandrea M.D., Mach K.J., Bilir T.E., Chatterjee M., Ebi K.L., Estrada Y.O., Genova R.C. (2014). Europe. Climate Change 2014: Impacts, Adaptation, and Vulnerability.

[5] Olesen J.E., Trnka M., Kersebaum K.C., Skjelvåg A.O., Seguin B., Peltonen-Sainio P., Rossi F., Kozyra J., Micale F. (2011). Impacts and adaptation of European crop production systems to climate change. Eur. J. Agron..

[6] Olesen J.E., Bindi M. (2002). Consequences of climate change for European agricultural productivity, land use and policy. Eur. J. Agron..

[7] Trnka M., Olesen J.E., Kersebaum K.C., Skjelvåg A.O., Eitzinger J., Seguin B., Peltonen-Sainio P., Rötter R., Iglesias A., Orlandini S. (2011). Agroclimatic conditions in Europe under climate change. Glob. Chang. Biol..

[8] Trnka M., Hlavinka P., Semenov M.A. (2015). Adaptation options for wheat in Europe will be limited by increased adverse weather events under climate change. J. R. Soc. Interface.

[9] Mäkinen H., Kaseva J., Balek J., Kersebaum K., Nendel C., Gobin A., Olesen J., Bindi M., Ferrise R., Moriondo M. (2018). Sensitivity of European wheat to extreme weather. Field Crop. Res..

[10] Prasad P.V.V., Maduraimuthu D. (2014). Response of floret fertility and individual grain weight of wheat to high temperature stress: Sensitive stages and thresholds for temperature and duration. Funct. Plant Biol..

[11] Porter J.R., Semenov M.A. (2005). Crop responses to climatic variation. Philos. Trans. R. Soc. Lond. B Biol. Sci..

[12] Porter J.R., Gawith M. (1999). Temperatures and the growth and development of wheat: A review. Eur. J. Agron..

[13] Luo Q. (2011). Temperature thresholds and crop production: A review. Clim. Chang..

[14] Barnabás B., Jäger K., Féhér A. (2008). The effect of drought and heat stress on reproductive processes in cereals. Plant Cell Environ..

[15] Jovanovic Z., Stikic R., Lee T.S. (2012). Strategies for Improving Water Productivity and Quality of Agricultural Crops in an Era of Climate Change. Irrigation Systems and Practices in Challenging Environments.

[16] Albrizio R., Todorovic M., Matic T., Stellacci A.M. (2010). Comparing the interactive effects of water and nitrogen on durum wheat and barley grown in a Mediterranean environment. Field Crop. Res..

[17] Boteta L. (2013). Gestão da rega do trigo. Gd. Cult..

[18] Patanita M., Tomaz A., Dôres J. (2019). Ainda a rega dos cereais de sementeira outono-invernal. Agrotec.

[19] Alghory A., Yazar A. (2018). Evaluation of net return and grain quality characteristics of wheat for various irrigation strategies under the Mediterranean climatic conditions. Agric. Water Manag..

[20] Acevedo E., Silva P.S., Silva H. (2002). Wheat Growth and Physiology. Bread Wheat. Improvement and Production.

[21] Pala M., Matar A., Mazid A. (1996). Assessment of the effects of environmental factors on the response of wheat to fertilizer in on-farm trials in a Mediterranean Type environment. Exp. Agric..

[22] Tomaz A., Patanita M., Guerreiro I., Boteta L., Ferro Palma J. (2018). Efficient use of water and nutrients in irrigated cropping systems in the Alqueva region. Span. J. Soil Sci..

[23] Liu W., Wang J., Wang C., Ma G., Wei Q., Lu H., Xie Y., Ma D., Kang G. (2018). Root growth, water and nitrogen use efficiencies in winter wheat under different irrigation and nitrogen regimes in North China Plain. Front. Plant Sci..

[24] Plaut Z., Butow B.J., Blumenthal C.S., Wrigley C.W. (2004). Transport of dry matter into developing wheat kernels and its contribution to grain yield under post-anthesis water deficit and elevated temperature. Field Crop. Res..

[25] Blandino M., Marinaccio F., Vaccino P., Reyneri A. (2015). Nitrogen Fertilization Strategies Suitable to Achieve the Quality Requirements of Wheat for Biscuit Production. Agron. J..

[26] Yu Z., Islam S., She M., Diepeveen D., Zhang Y., Tang G., Zhang J., Juhasz A., Yang R., Ma W. (2018). Wheat grain protein accumulation and polymerization mechanisms driven by nitrogen fertilization. Plant J..

[27] Trenkel M.E. (1997). Improving Fertilizer Use Efficiency. Controlled-Release and Stabilized Fertilizers in Agriculture.

[28] Chen D., Suter H.C., Islam A., Edis R., Freney J., N Walker C. (2008). Prospects of improving efficiency of fertiliser nitrogen in Australian agriculture: A review of enhanced efficiency fertilisers. Aust. J. Soil Res..

[29] Trenkel M.E. (2007). Special Fertilizers. Ullmann’s Agrochemicals.

[30] Arrobas M., Parada M., Magalhães P., Rodrigues M. (2011). Nitrogen-use efficiency and economic efficiency of slow-release N fertilisers applied to irrigated turfs in a Mediterranean environment. Nutr. Cycl. Agroecosyst..

[31] Maharjan B., Venterea R., Rosen C. (2014). Fertilizer and Irrigation Management Effects on Nitrous Oxide Emissions and Nitrate Leaching. Agron. J..

[32] Tomaz A., Ferro Palma J., Guerreiro I., Patanita M.I., Penacho J., Dôres J., Costa M.N., Rosa E., Patanita M. (2017). An Overview on the Use of Enhanced Efficiency Nitrogen Fertilizers in Irrigated Mediterranean Agriculture. BJSTR.

[33] Porter J.R., Xie L., Challinor A.J., Cochrane K., Howden S.M., Iqbal M.M., Lobell D.B., Travasso M.I., Field C.B., Barros V.R., Dokken D.J., Mach K.J., Mastrandrea M.D., Bilir T.E., Chatterjee M., Ebi K.L., Estrada Y.O., Genova R.C. (2014). Food Security and Food Production Systems. Climate Change 2014: Impacts, Adaptation, and Vulnerability.

[34] Ventrella D., Charfeddine M., Moriondo M., Rinaldi M., Bindi M. (2012). Agronomic adaptation strategies under climate change for winter durum wheat and tomato in southern Italy: Irrigation and nitrogen fertilization. Reg. Environ. Chang..

[35] Raza A., Razzaq A., Mehmood S., Zou X., Zhang X., Lv Y., Xu J. (2019). Impact of Climate Change on Crops Adaptation and Strategies to Tackle Its Outcome: A Review. Plants.

[36] Paymard P., Bannayan M., Haghighi R.S. (2018). Analysis of the climate change effect on wheat production systems and investigate the potential of management strategies. Nat. Hazards.

[37] Carvalho M., Serralheiro R., Corte-Real J., Valverde P. (2015). Implications of climate variability and future trends on wheat production and crop technology adaptations in southern regions of Portugal. Water Util. J..

[38] Tadayon M.R., Ebrahimi R., Tadayyon A. (2012). Increased Water Productivity of Wheat under Supplemental Irrigation and Nitrogen Application in a Semi-arid Region. J. Agric. Sci. Technol..

[39] Zhang X., Qin W., Chen S., Shao L., Sun H. (2017). Responses of yield and WUE of winter wheat to water stress during the past three decades—A case study in the North China Plain. Agric. Water Manag..

[40] Tomaz A., Patanita M., Guerreiro I., Boteta L., Palma J.F. (2017). Water use and productivity of maize-based cropping systems in the Alqueva region (Portugal). Cereal Res. Commun..

[41] Nasseri A., Ali Fallahi H., Siadat A., Eslami-Gumush Tappeh K. (2009). Protein and N-use efficiency of rainfed wheat responses to supplemental irrigation and nitrogen fertilization. Arch. Agron. Soil Sci..

[42] Fallahi H., Nasseri A., Siadat A. (2008). Wheat Yield Components are Positively Influenced by Nitrogen Application under Moisture Deficit Environments. Int. J. Agric. Biol..

[43] Ul-Allah S., Iqbal M., Maqsood S., Naeem M., Ijaz M., Ashfaq W., Hussain M. (2018). Improving the performance of bread wheat genotypes by managing irrigation and nitrogen under semi-arid conditions. Arch. Agron. Soil Sci..

[44] Garofalo P., Ventrella D., Kersebaum K.C., Gobin A., Trnka M., Giglio L., Dubrovský M., Castellini M. (2019). Water footprint of winter wheat under climate change: Trends and uncertainties associated to the ensemble of crop models. Sci. Total Environ..

[45] Ruiz-Ramos M., Mínguez M. (2010). Evaluating uncertainty in climate change impacts on crop productivity in the Iberian Peninsula. Clim. Res..

[46] Palosuo T., Kersebaum K.C., Angulo C., Hlavinka P., Moriondo M., Olesen J.E., Patil R.H., Ruget F., Rumbaur C., Takáč J. (2011). Simulation of winter wheat yield and its variability in different climates of Europe: A comparison of eight crop growth models. Eur. J. Agron..

[47] IPMA (Instituto Português do Mar e da Atmosfera) (2017). Boletim Climatológico Anual de Portugal Continental 2017.

[48] Palmer W.C. (1965). Meteorological Drought.

[49] Costa N., Bagulho A., Patanita M. (2017). Qualidade dos Trigos. Parte III/III: Fatores que afetam a qualidade do trigo. Gd. Culturas.

[50] Oliveira P., Patanita M., Dôres J., Boteta L., Palma J.F., Patanita M.I., Guerreiro I., Penacho J., Costa M.N., Rosa E. (2019). Combined effects of irrigation management and nitrogen fertilization on soft wheat productive responses under Mediterranean conditions. E3S Web Conf..

[51] ANPOC (Associação Nacional de Produtores de Oleaginosas, Proteaginosas e Cereais), LVR (Lista de Variedades Recomendadas) (2017). Lista de Variedades Recomendadas de Trigo Mole.

[52] IPMA (Instituto Português do Mar e da Atmosfera) (2019). Normais Climatológicas.

[53] IUSS (2014). Working Group WRB World Reference Base for Soil Resources 2014, Update 2015.

[54] SROA—Serviço de Reconhecimento e Ordenamento Agrário (1961). Carta de Solos de Portugal.

[55] Virmani S.M., Sahrawat K.L., Burford J.R. Physical and Chemical Properties of Vertisols and their Management. Proceedings of the Twelfth International Congress of Soil Science.

[56] COTR (Centro Operativo e de Tecnologia do Regadio) (2018). SAGRA—Sistema Agrometeorológico para a Gestão da Rega no Alentejo. http://www.cotr.pt/servicos/sagranet.php.

[57] COTR (Centro Operativo e de Tecnologia do Regadio) (2018). MOGRA—Modelo de Gestão da Rega para o Alentejo. http://www.cotr.pt/servicos/mogra.php.

[58] Allen R.G. (1998). , Pereira L.S., Raes D., Smith M. Crop Evapotranspiration—Guidelines for Computing Crop Water Requirements.

[59] Zadoks J.C., Chang T.T., Konzak C.F. (1974). A decimal code for the growth stages of cereals. Weed Res..

[60] (2012). Ministério da Agricultura, do Mar, do Ambiente e do Ordenamento do Território. Ordinance 259/2012 that Establishes the Action Program for Areas Vulnerable to Nitrates from Agricultural Sources.

[61] (1997). Ministério do Ambiente. Decree-Law 235/97 Implementing Council Directive 91/676/EEC of Concerning the Protection of Waters Against Pollution Caused by Nitrates from Agricultural Sources.

[62] EEC (1991). The Nitrates Directive—Council Directive Concerning the Protection of Waters against Pollution Caused by Nitrates from Agricultural Sources.

[63] Dias P. (2003). Monitorização de Água no Solo: Sonda Profile Probe PR1.

[64] Sentek (2011). Calibration Manual for Sentek Soil Moisture Sensors.

